# Clinical and Molecular Findings in Mendelian Susceptibility to Mycobacterial Diseases: Experience From India

**DOI:** 10.3389/fimmu.2021.631298

**Published:** 2021-02-25

**Authors:** Prasad D. Taur, Vijaya Gowri, Ambreen Abdulwahab Pandrowala, Vaishnavi V. Iyengar, Akshaya Chougule, Zainab Golwala, Shraddha Chandak, Reepa Agarwal, Purva Keni, Neha Dighe, Minnie Bodhanwala, Shakuntala Prabhu, Biju George, N. A. Fouzia, Eunice Sindhuvi Edison, Arun Kumar Arunachalam, Manisha Rajan Madkaikar, Aparna Dhondi Dalvi, Reetika Malik Yadav, Umair Ahmed Bargir, Priyanka Madhav Kambli, Amit Rawat, Jhumki Das, Vibhu Joshi, Rakesh Kumar Pilania, Ankur Kumar Jindal, Sunil Bhat, Sagar Bhattad, Jeeson Unni, Nita Radhakrishnan, Revathi Raj, Ramya Uppuluri, Shivani Patel, Harsha Prasada Lashkari, Amita Aggarwal, Manas Kalra, Zarir Udwadia, Vibha Sanjay Bafna, Tarun Kanade, Anne Puel, Jacinta Bustamante, Jean Laurent Casanova, Mukesh M. Desai

**Affiliations:** ^1^Department of Immunology, B. J. Wadia Hospital for Children, Mumbai, India; ^2^Department of Clinical Hematology, Christian Medical College, Vellore, India; ^3^Indian Council of Medical Research-National Institute of Immunohematology, Mumbai, India; ^4^Department of Pediatrics, Advanced Pediatrics Centre, Post Graduate Institute of Medical Education and Research, Chandigarh, India; ^5^Mazumdar Shaw Cancer Centre, Narayana Health City, Bengaluru, India; ^6^ASTER CMI Hospitals, Bengaluru, India; ^7^Superspeciality Hospital, Noida, India; ^8^Apollo Hospitals, Chennai, India; ^9^Kasturba Medical College and Hospital, Mangalore, India; ^10^Department of Clinical Immunology and Rheumatology, Sanjay Gandhi Post Graduate Institute of Medical Sciences, Lucknow, India; ^11^Sir Ganga Ram Hospital, New Delhi, India; ^12^Hinduja Hospital, Mumbai, India; ^13^Bharati Hospital, Pune, India; ^14^Rainbow Safalya Hospital, Nashik, India; ^15^University of Paris, Institute Imagine, INSERM, Paris, France; ^16^Laboratory of Human Genetics of Infectious Diseases, Necker Branch, INSERM UMR1163, Paris, France; ^17^St. Giles Laboratory of Human Genetics of Infectious Diseases, Rockefeller Branch, The Rockefeller University, New York, NY, United States; ^18^Study Center for Immunodeficiencies, Necker Hospital for Sick Children, AP-HP, Paris, France; ^19^Howard Hughes Medical Institute, New York, NY, United States

**Keywords:** IL-12/IL-23/ISG15/IFN-γ axis, intracellular pathogens, BCG-osis, *Mycobacterium tuberculosis* complex, IL-12Rβ1 defect, anti-tubercular treatment

## Abstract

Mendelian Susceptibility to Mycobacterial diseases (MSMD) are a group of innate immune defects with more than 17 genes and 32 clinical phenotypes identified. Defects in the IFN-γ mediated immunity lead to an increased susceptibility to intracellular pathogens like mycobacteria including attenuated *Mycobacterium bovis*-Bacillus Calmette-Guérin (BCG) vaccine strains and non-tuberculous environmental mycobacteria (NTM), *Salmonella*, fungi, parasites like *Leishmania* and some viruses, in otherwise healthy individuals. Mutations in the *IL12RB1* gene are the commonest genetic defects identified. This retrospective study reports the clinical, immunological, and molecular characteristics of a cohort of 55 MSMD patients from 10 centers across India. Mycobacterial infection was confirmed by GeneXpert, Histopathology, and acid fast bacilli staining. Immunological workup included lymphocyte subset analysis, Nitro blue tetrazolium (NBT) test, immunoglobulin levels, and flow-cytometric evaluation of the IFN-γ mediated immunity. Genetic analysis was done by next generation sequencing (NGS). Disseminated BCG-osis was the commonest presenting manifestation (82%) with a median age of presentation of 6 months due to the practice of BCG vaccination at birth. This was followed by infection with *Salmonella* and non-typhi *Salmonella* (13%), *Cytomegalovirus (CMV)* (11%), *Candida* (7%), NTM (4%), and *Histoplasma* (2%). Thirty-six percent of patients in cohort were infected by more than one organism. This study is the largest cohort of MSMD patients reported from India to the best of our knowledge and we highlight the importance of work up for IL-12/IL-23/ISG15/IFN-γ circuit in all patients with BCG-osis and suspected MSMD irrespective of age.

## Introduction

Mendelian Susceptibility to Mycobacterial diseases (MSMD) now also known as Inborn Errors of IFN-γ immunity (IEI) are a group of innate or intrinsic immune defects localized to 17 genes and 32 clinical phenotypes identified (1–3). IL-12/23/ISG15-IFN-γ axis is the principal immunological pathway for intra-macrophagic pathogens (4, 5). Defects in this pathway lead to an increased susceptibility to intracellular pathogens like mycobacteria including attenuated *Mycobacterium bovis*- Bacillus Calmette-Guérin (BCG) vaccine strains and non-tuberculous environmental mycobacteria (EM), *Salmonella*, fungi, parasites like *Leishmania*, and some viruses, in otherwise healthy individuals (4, 6–9). Tuberculosis is the commonest public health problem in India with an estimated incidence of 2.4 million cases in 2019 (10). BCG vaccination is universally administered to all babies soon after birth (11), to protect against severe forms of tuberculosis. Adverse event following BCG immunization might be the presenting manifestation in MSMD.

In a setting of strong clinical suspicion, flow-cytometric evaluation of IL-12/23-IFN-γ pathway followed by molecular work-up for identifying the genetic etiology is warranted. Our first patient of IL-12Rβ1 defect was diagnosed with help from Dr. Dinakantha Kumararatne from Cambridge. Subsequently, our initial cases suspected with MSMD were evaluated by Dr. Jacinta Bustamante and Pr. Jean-Laurent Casanova at Paris; and a genetic cause could be identified in nine patients with three IL-12Rβ1 complete defects, two IFN-γR1 (partial dominant), two STAT1 (partial dominant, loss-of-function), one complete IFN-γR1 defect, and one complete IL-12p40 defect. In India, with increasing awareness about MSMD, pediatricians, infectious disease specialists, hemato-oncologists and those dealing with the menace of *Mycobacterium tuberculosis* (*M. tb*) started recognizing and appreciating genetic factors responsible for susceptibility to mycobacterial tuberculosis. With the ease of access to next generation sequencing (NGS) in recent years, the diagnosis of MSMD has increased.

There is a paucity of literature on MSMD from India with only a few published case reports (12–15). In this study, we report clinical, immunological, and molecular characteristics of a retrospective cohort of 55 MSMD patients from 10 centers across India.

## Materials and Methods

Ten participating of Primary immunodeficiencies (PID) centers from India contributed data for this retrospective analysis. The participating centers included Bai Jerbai Wadia Hospital for Children, Mumbai (*n* = 14), Christian Medical College; Vellore (*n* = 11), Indian Council of Medical Research—National Institute Immunohaematology; Mumbai (*n* = 10), ACPED (Advance Center for Pediatrics) PGI, Chandigarh (*n* = 8), ASTER CMI; Bangalore (*n* = 3), Narayana Hrudayalaya; Bangalore (*n* = 3), Kasturba Medical College and Hospital; Mangalore (*n* = 2), Apollo Hospital; Chennai (*n* = 2), Superspeciality Pediatric Hospital and Post Graduate Teaching Institute; Noida (*n* = 1), and Sir Ganga Ram Hospital, Delhi (*n* = 1). All centers were contacted *via* email and requested to provide details of their MSMD patients on a pre-designed Microsoft Excel datasheet. Details included demographic information, clinical manifestations, family history, microbiological and immunological investigations, genetic evaluation, management, and follow-up. Being a retrospective analysis of data, it was exempted from ethics approval.

Diagnosis of infection with *Mycobacterium tuberculosis* complex (MTBC) was confirmed by PCR in addition to histopathologic and microbiologic findings of acid-fast bacilli (AFB) detected from lymph nodes or broncho-alveolar lavage. Diagnostic approach for suspected MSMD included basic lymphocyte subset analysis, nitroblue tetrazolium (NBT) test, immunoglobulin levels, and flow-cytometric evaluation of the IFN-γR1 (CD119), IL-12Rβ1 (CD212), serum IFN-γ levels, STAT1, and STAT4 phosphorylation along with NGS for making a molecular diagnosis. NGS included PID targeted-gene panel and clinical Exome sequencing. Imaging studies including chest X-ray, ultrasound, and chest CT scan were used when internal organ involvement was suspected. The specialized assays were used for rapid identification of the candidate gene when molecular facilities were not freely available initially. Eventually with the easy availability of NGS facilities, the relevant assays were performed for functional validation of the identified mutation and has been carried out in majority of the cases. Some centers performed molecular workup directly after the basic immune work-up for the patients suspected with MSMD.

Statistical analysis of data was obtained on a predesigned worksheet (Excel, Microsoft Office) and analyzed using Graph pad Prism (Chicago, IL, USA) for Microsoft Windows. Kaplan Meier's analysis was used to estimate the risk of BCG related complications.

## Results

### Patient Characteristics

**Figure 1:**
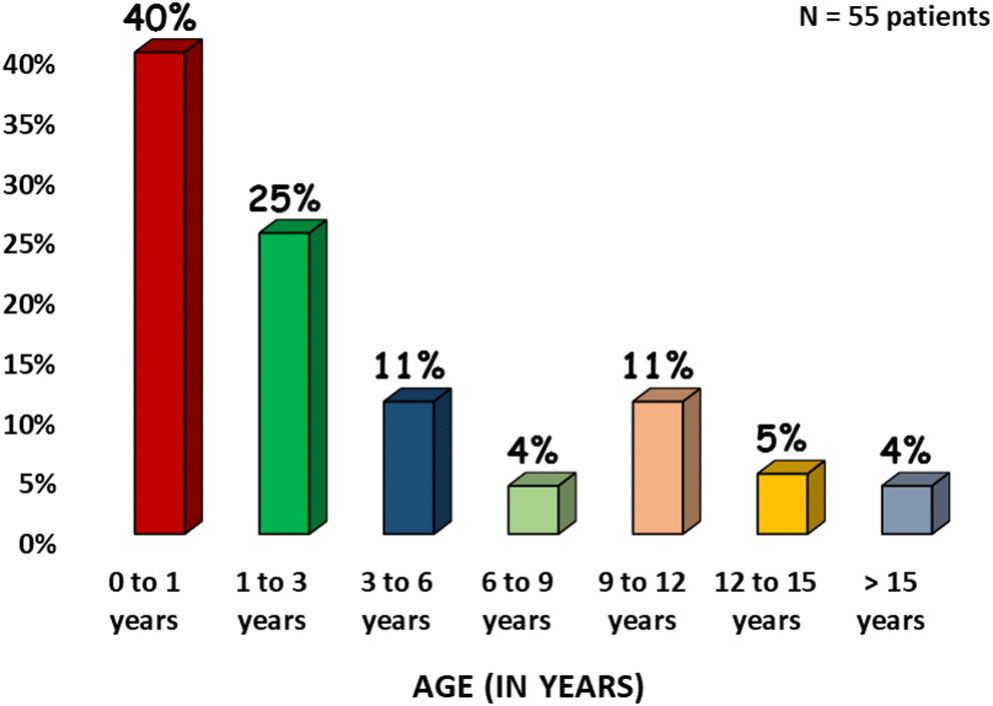
Age-wise distribution at presentation.

In this study, we analyzed a total of 55 patients of MSMD of which 51 (93%) had a confirmed molecular diagnosis and four were evaluated by flow cytometry. The clinical and demographic details of the patients are presented in Table 1. There was a slight male preponderance with 56% males and 44% females. History of consanguinity was present in 58% families and 33% families had history of a previous affected sibling. The median age of presentation was 6 months (0.5–300 months). Figure 1 shows the age-wise distribution at presentation.

**Table 1:** Demographic and clinical findings.

**Patient ID**	**Age of presentation (in months)**	**Sex**	**Consanguinity**	**Family history**	**BCGosis**	**Salmonella**	**Other organisms**	**Type of MSMD Defect**	**Outcome**
P1	0.5	F	+	+	+	–	*CMV*	*IFN-γR1*	D
P2	6	F	–	–	+	–	*Pseudomonas aeruginosa, CMV*	*IFN-γR1*	A
P3	3	M	+	–	+	–	–	*IL-12p40*	A
P4	6	F	+	–	+	–	–	*STAT1*	D
P5	3	F	+	+	+	–	–	*STAT1*	D
P6	11	M	+	–	+	–	–	*IFN-γR2*	A
P7	168	M	+	+	+	–	*Giardia, Histoplasma*	*IL-12Rβ1*	A
P8	120	M	–	+	+	+	–	*IL-12Rβ1*	A
P9	300	F	+	–	–	+	–	*IL-12Rβ1*	A
P10	18	M	+	–	+	–	*Candida*	*IL-12Rβ1*	D
P11	120	F	–	–	+	–	*Staphylococcus aureus, NTM, Molluscum* (*Coxsackie*)	*IFN-γR1*	A
P12	24	M	+	+	+	–	–	*IFN-γR1*	D
P13	180	M	+	–	–	–	*Mycobacterium leprae*	*IFNγR1*	D
P14	9	F	–	–	+	–	–	*IL-12Rβ1*	A
P15	27	F	–	–	+	–	–	*IL-12Rβ1*	Lost F/U
P16	48	M	–	–	+	–	*Candida*	*IL-12Rβ1*	Lost F/U
P17	12	F	+	+	+	–	–	*IL-12Rβ1*	A
P18	3	F	+	+	+	–	–	*IL-12Rβ1*	D
P19	132	M	+	–	+	–	–	*IL-12Rβ1*	A
P20	14	F	+	–	+	–	–	*IFN-γR2*	A
P21	5	F	+	+	+	–	–	*IFN-γR1*	Lost F/U
P22	3	M	+	–	+	–	–	*IFN-γR1*	Lost F/U
P23	4	F	–	–	+	–	–	*IFN-γR1*	A
P24	4	M	+	–	+	–	–	*IFN-γR2*	D
P25	2	F	+	–	+	–	–	*IL-12Rβ1*	D
P26	6	F	+	–	–	–	*Candida*	*RORC*	A
P27	72	F	–	–	+	–	*Varicella Zoster*	*IFNγR1*	A
P28	4	F	+	+	+	–	–	*IL-12Rβ1*	A
P29	3	M	+	+	+	–	–	*IFN-γR2*	D
P30	6	M	–	+	+	–	–	*STAT1*	A
P31	6	M		–	+	–	–	*IFNγR1*	Lost F/U
P32	7	F	+	–	+	+	–	*IL-12Rβ1*	A
P33	4	M	–	–	+	–	–	*IL-12Rβ1*	A
P34	2	F	–	–	+	–	–	*IL-12Rβ1*	Lost F/U
P35	13	M	+	–	+	–	*CMV, NTM*	*IFN-γR1*	A
P36	3	M	+	–	+	–	–	*IL-12Rβ1*	A
P37	3	M	+	–	+	–	*Streptococcus pneumoniae, Enterococcus, BOCA VIRUS*	*ISG15*	D
P38	3	M	–	–	+	–	–	*IFN-γR2*	D
P39	4	F	–	+	–	+	–	*IL-12Rβ1*	A
P40	1	F	–	–	+	–	*Staphylococcus aureus*	*IL-12Rβ1*	A
P41	1	M	–	–	+	–	–	*IL-12Rβ1*	D
P42	1	M	–	–	–	–	–	*STAT1*	A
P43	7	M	–	+	+	–	–	*IFN-γR2*	Lost F/U
P44	48	M	–	–	+	+	–	*IFN-γR1*	Lost F/U
P45	120	F	–	–	–	+	*Proteus*	*IL-12Rβ1*	Lost F/U
P46	120	M	–	+	+	+	*Clostridium difficile*	*IL-12Rβ1*	Lost F/U
P47	96	M	–	–	+	–	–	*IFNγR1*	Lost F/U
P48	36	F	+	–	+	–	*Streptococcus pyogenes*	*IFNγR1*	Lost F/U
P49	6	F	+	+	+	–	–	*IL-12Rβ1*	Lost F/U
P50	6	M	–	+	+	–	–	*IL-12Rβ1*	Lost F/U
P51	9	M	+	+	–	–	*CMV*	*IFN-γR2*	A
P52	6	M	+	+	–	–	*Neisseria, CMV*	*IFN-γR2*	D
P53	6	M	+	–	–	–	*Candida*	*IL-12Rβ1*	A
P54	3	M	–	–	+	–	*CMV*	*IFN-γR1*	A
P55	60	M	–	–	–	–	–	*IL-12Rβ1*	A

*D, dead; A, alive; F/U, follow-up*.

**Figure 2:**
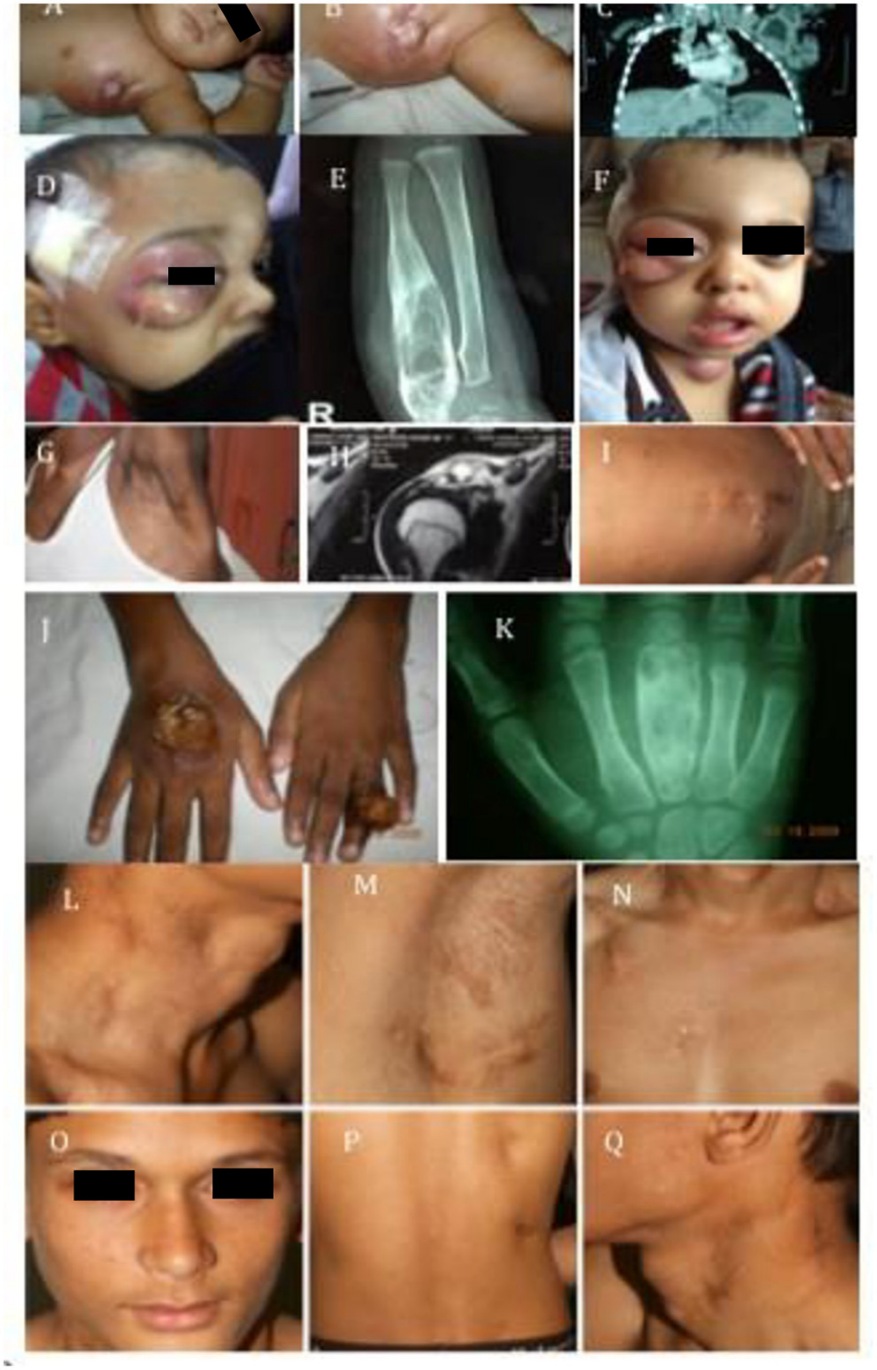
Clinical images. **(A–C)** Show image of BCG-osis without failure to thrive: IL-12Rβ1 defect. **(D–F,J,K)** Represent multi-focal bone involvement seen in PD-IFNγR1 defect or AD-STAT1 defect. **(G–I)** Shows child with BCG-osis, shoulder, and disseminated *Histoplasmosis*: IL-12Rβ1 defect. **(L–Q)** Represent another case of BCG-osis with impetigo and lymphadenopathy whose culture grew non-typhi *Salmonella*: IL-12Rβ1 defect.

The commonest clinical presentation was disseminated BCG-osis (82% patients) [ESID criteria for BCG-itis and BCG-osis was followed (16)], representative clinical images are shown in Figure 2. The average number of organs involved in patients with BCG-osis was three.

### Microbiological Spectrum

**Figure 3:**
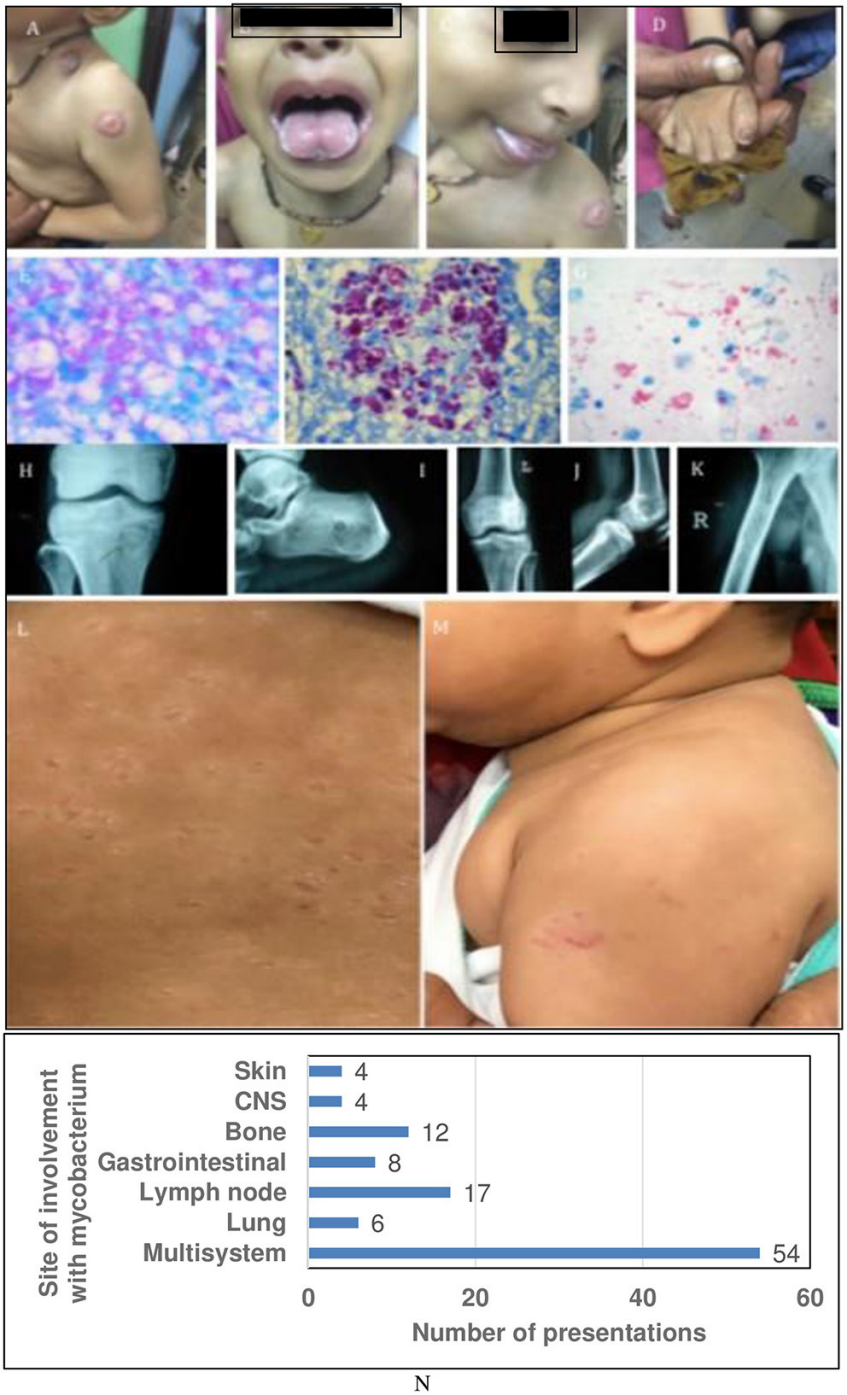
Representative clinical images and sites of involvement of Mycobacteria. **(A–D)** Show BCG-osis with candidiasis: IL-12Rβ1 defect or ROR-γT defect. **(E–G)** Show exuberant growth of AFB in tissue Liver Biopsy **(F)** also shows *CMV* inclusions. **(H–K)** multifocal bone TB: IFN-γR1 or IFN-γR2 defect. **(L,M)** Show cutaneous BCG-osis. **(N)** Showing graph with sites of involvement with mycobacterium.

All patients in the cohort had mycobacterial disease with MTBC (96%), and non-tuberculous mycobacteria (NTM) in (4%). Multisystem involvement with mycobacteria was the commonest followed by lymph node involvement, tubercular osteomyelitis, pulmonary, skin, and central nervous system (CNS) in the order of decreasing frequency (Figure 3).

**Figure 4:**
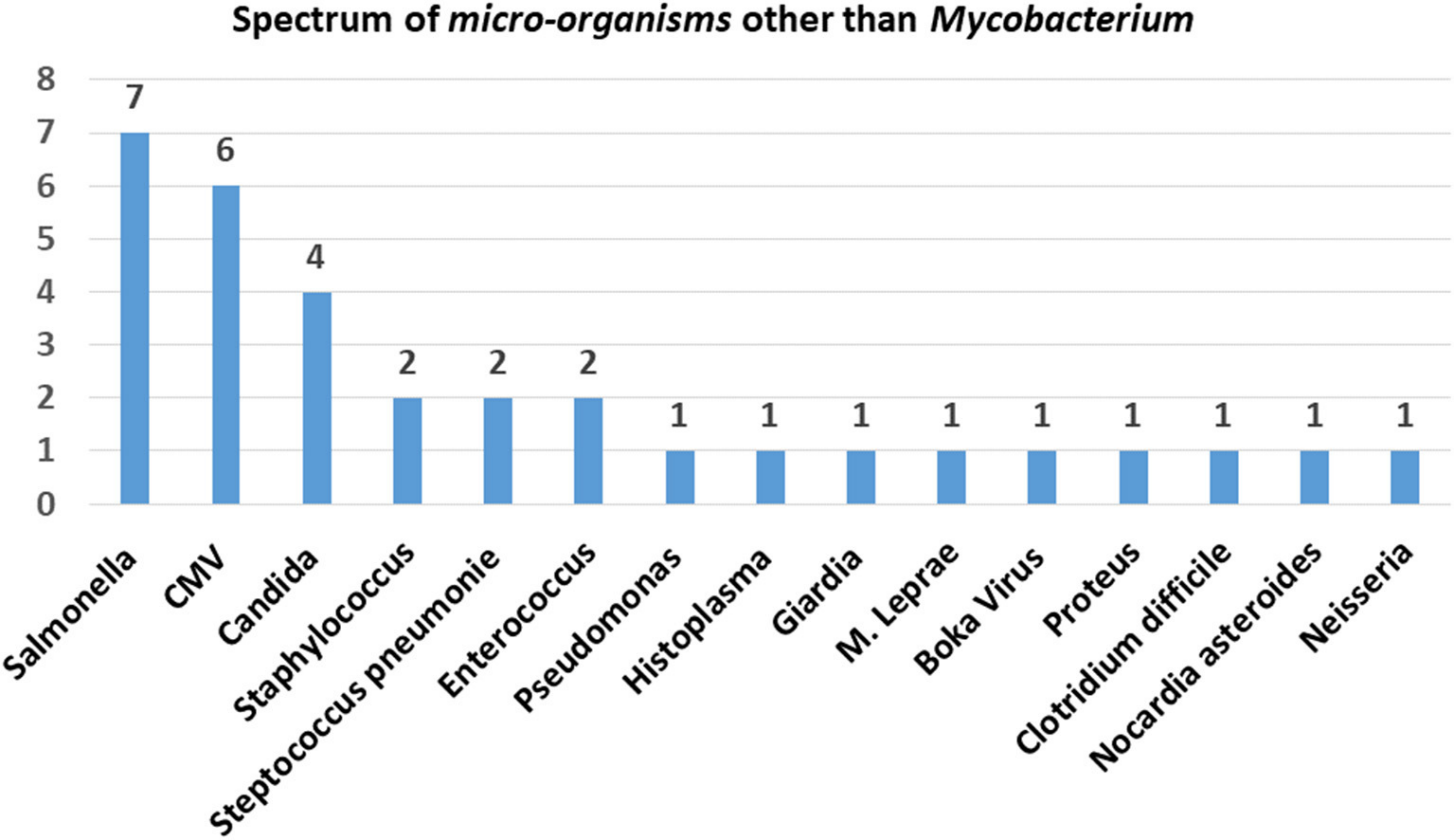
Microbiological spectrum.

Thirty-six percent of patients in cohort were infected by more than one organism. Other infections associated with our cohort were *Salmonella* and non-typhi *Salmonella* (13%), *Candida* (7%), *Histoplasma* (2%), *Nocardia* (2%), *Mycobacterium leprae* (2%) other bacterial infections like *Staphylococcus aureus* (4%), Streptococci (4%), *Pseudomonas aeruginosa* (2%), *Neisseria* (2%), *Proteus mirabilis* (2%), *Clostridium difficile* (2%), *Enterococci* (4%), and viruses *Cytomegalovirus* (CMV, 11%), Molluscum (2%), Varicella zoster (2%), *Bocavirus* (2%). The overall microbiological spectrum and distribution of infections with different families of micro-organisms are presented in Figure 4.

### Immunological and Molecular Evaluation

Evaluation of the IL-12/23/IFN-γ pathway by flow-cytometry could be performed in 21 patients and the results were consistent with the underlying molecular defect. NGS analysis in 55 MSMD patients within 51 families led to the identification of 5 previously reported and 24 novel mutations in 7 different genes as presented in Table 2. The pathogenicity of novel mutations identified in our cohort was determined by *in silico* tools like Mutation Taster, SIFT and Polyphen-2, and genotype-phenotype correlation.

**Table 2:** Molecular findings.

**Gene**	**Defect**	**Number of patients**	**Patient ID**	**Position**	**Nucleotide change**	**Amino acid change**	**Zygosity**	**Mutation type**	**Reported/novel**
*IL12R*β1	Complete	1	P7	Exon 9	c.982_982delC	p.M328Cfs^*^41	Homozygous	Deletion	Novel
		2	P9, P25	Exon 16	c.1786A>G	p.K596E	Homozygous	Missense	rs567051378 (Reported Benign in Clinvar)
		11	P10, P15, P19, P28, P32, P34 P39, P40, P41, P46, P50	Exon 9	c.962C>A	p. S321^*^	Homozygous	Nonsense	rs147766868 (PMID 30255293) (17)
		6	P8, P16, P17, P18, P45, P33	Intron 15	c.1791+2T>G		Homozygous	Essential splicing site	rs554063682 (PMID 26976630) (17, 18)
		2	P36, P55	Exon 7	c.698_698delC	p.P233Lfs^*^9	Homozygous	Deletion	Novel
		1	P14	Exon 8	c.599del	p.L200Rfs^*^3	Homozygous	Deletion	Novel (rs1169002203)
		1	P49	Intron 14	c.1738+2 T>A		Homozygous	Essential splicing site	Novel
		1	P53	Exon 6	c.511C>T	p.Q171^*^	Homozygous	Nonsense	Novel
*IFNGR1*	Partial	4	P2, P11, P44, P47	Exon 6	c.818_821 delTTAA	p.N274Hfs^*^2	Heterozygous	Deletion	Novel
	Complete	1	P12	Exon 5	c.601_601delC	p.Q201Sfs^*^2	Homozygous	Deletion	Novel
		1	P1	Exon 6	c.838_839dupA	p.S280Kfs^*^3	Homozygous	Duplication	Novel
		1	P48	Exon 3	c.328G>T	p.E110^*^	Homozygous	Nonsense	Novel
		1	P21	Exon 4	c.389C>A	p.P130H	Homozygous	Missense	Novel
		1	P35	Exon 2	c.110 T>C	p.I37T	Homozygous	Missense	rs945137618 (PMID 28744922) (19)
		1	P31	Exon 5	c.653_655del	p.E218del	Homozygous	In-frame deletion	rs587776858 (PMID 10811850) (20)
		1	P54	Exon 7	c.1068delG	p.T357Lfs^*^15	Homozygous	Deletion	Novel
*IFNGR2*	Complete	1	P6	Exon 2	c.196C>T	p.Q66^*^	Homozygous	Nonsense	Novel
		1	P20	Intron 4	c.561+1G>A		Homozygous	Essential splicing site	Novel
		1	P24	Exon 4	c.540G>A	p.W180^*^	Homozygous	Nonsense	Novel
		1	P29	Exon 6	c.782_790del TGCTGGCAG	p.V261_A263del	Homozygous	In frame deletion	Novel
		1	P38	Exon 4	c.488T>C	p.F163S	Homozygous	Missense	Novel
		1	P43	Intron 3	c.412+2T>A		Homozygous	Essential splicing site	Novel
		2	P51, P52	Intron 2	c.207-1G>A		Homozygous	Essential splicing site	Novel
*ISG15*	Complete	1	P37	Exon 2	c.454_455delCT	p.L152Afs^*^?	Homozygous	Deletion	Novel
*STAT1*	Complete	2	P4, P5	Exon 9	c.769dup	p.D257Gfs^*^22	Homozygous	Duplication	Novel
	Partial	1	P30	Exon 9	c.749G>A	p.G250E	Heterozygous	Missense	Novel
		1	P42	Intron 20	c.1728-4C>T		Heterozygous	Essential splicing site	Novel (rs760805208)
*IL12B*		1	P3	Exon 4	c.429G>A	p.W143^*^	Homozygous	Nonsense	Novel (rs751288779)
*RORC*	Complete	1	P26	Exon 5	c.558T>G	p.Y186^*^	Homozygous	Nonsense	Novel

* *means termination; ? means unknown*.

**Figure 5:**
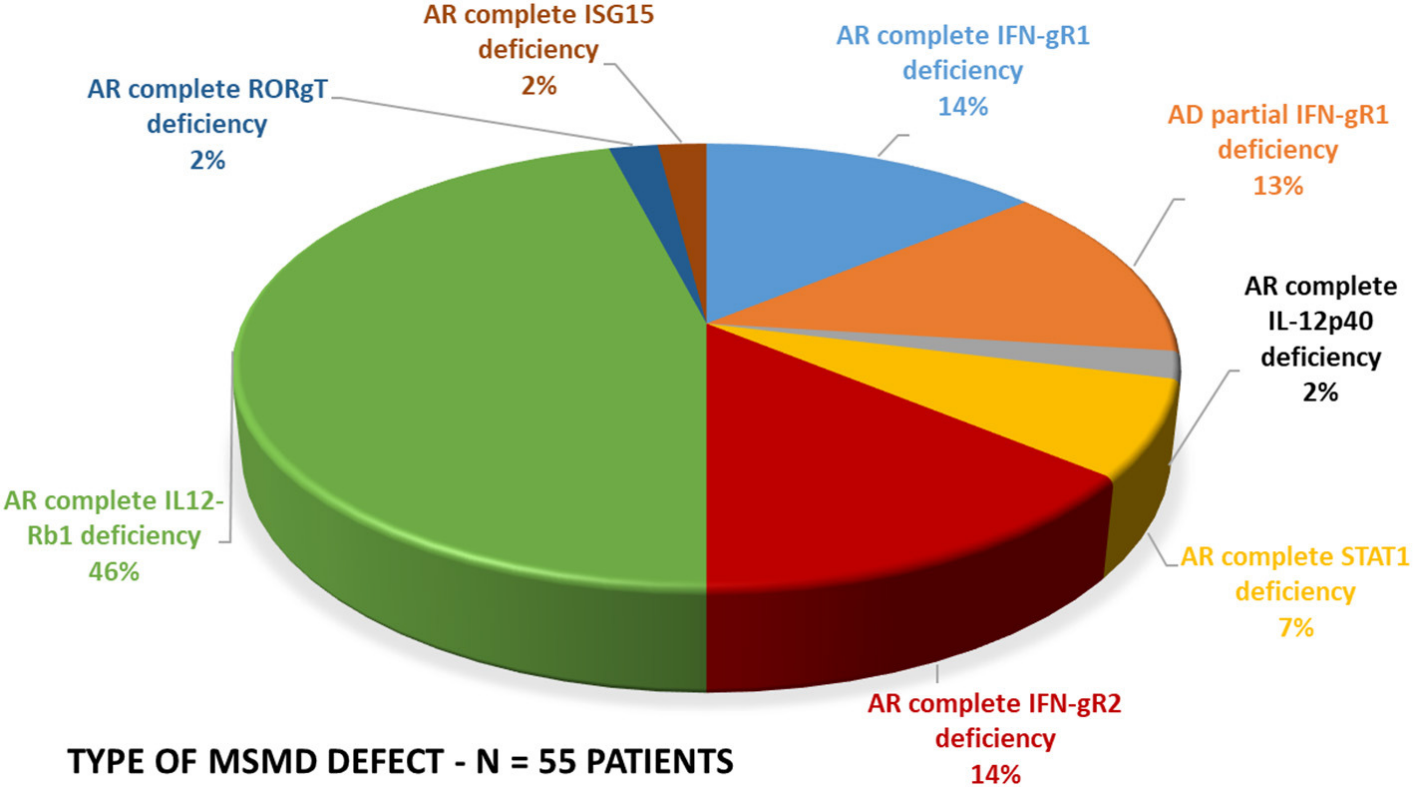
Molecular defects. The molecular defects identified in our cohort were IL-12Rβ1 (46%) followed by complete IFN-γR1 (14%), complete IFN-γR2 (14%), PD-IFN-γR1 (13%), complete STAT1 defect (7%) and IL-12p40 defect (2%), RORC(2%), and ISG15(2%).

The spectrum of molecular defects identified in the patients is presented in Figure 5. The prevalence of different molecular defects in India is consistent with world literature as shown in Figure 5 (21, 22).

#### AR Complete IL-12Rβ1 Deficiency

The complete IL-12Rβ1 defect was identified in 25 patients presenting at a median age of presentation of 9 months (Range 1–300 months). 21/25 (84%) patients presented with BCG-osis; Six patients (P8, P9, P32, P39, P45, and P46) had recurrent *Salmonella* infection and three patients (P10, P16, and P53) had *Candidiasis* along with tuberculosis. Nineteen patients (76%) had only BCG-osis and did not develop any other infections during the period of follow up. Autoimmune manifestations in the form of inflammatory bowel disease was seen in three patients (P8, P45, and P46) and one patient (P46) had autoimmune hemolytic anemia, leukocytoclastic vasculitis and was ANA positive. The other infections identified included *Histoplasma* (P7), *Giardia* (P7), *Staphylococcus* (P40), *Proteus* (P45), *Enterococcus* (P45), and *Clostridium difficile* (P46). One patient (P7) with histoplasmosis and BCG-osis also had CD4 lymphopenia and portal vein thrombosis. Consanguinity was present in 12/25(48%) patients and there was history of a previous affected sibling in 7/25(28%) families. Flow cytometric evaluation of IL-12Rβ1 expression was found to be absent in 11 patients where it was evaluated.

IL-12Rβ1 was the predominant gene affected in our cohort with a homozygous mutation located in exon 9, c.962C>A being the commonest mutation identified (11/25) followed by another mutation Intron 15 c.1791+2T>G (6/25). Two of our patients had homozygous missense mutation in exon 16 of *IL12R*β*1*, c.1786A>G. This mutation was reported as benign in Clinvar, however flow cytometry confirmed deficient *IL-12R*β*1*. Carrier status in parents was confirmed in three families. All patients received anti-mycobacterial treatment with antibiotics. Two siblings also had an aspiration of pus for BCG-itis. Three patients of complete IL-12Rβ1 deficiency (P32, P53, and P55) underwent allogeneic hematopoietic stem cell transplantation (HSCT), which engrafted well and the patients are doing well. P32 and P53 had father as haploidentical donor while P55 had a fully matched sibling donor. Four (16%) patients (P10, P18, P25, and P41) expired.

Three of our patients developed inflammatory bowel disease (IBD) during follow up which is not reported in world literature. IL-12Rβ1 defect also results in TH17 pathway defect that can be associated with autoimmunity and auto inflammation which could be the cause of IBD in our patients. This needs to be investigated further.

#### AR Complete IL-12p40 Deficiency

IL-12p40 defect was identified in one patient (P3) who presented at three months of age with BCG-osis and also needed admission twice due to tubercular pleural effusion. The patient received ATT thrice and is doing well.

#### AR Complete IFN-γR1 Deficiency

Complete IFN-γR1 deficiency was identified in eight patients. The median age of presentation was 5.5 months (0.5–36 months). BCG-osis was the presenting manifestation in all patients. Additional infections with *Streptococcus* (P48), and CMV (P1, P35, and P54) were observed. Flow cytometric evaluation of IFN-γR1 was performed in four patients and found to be absent in three. One patient (P1) revealed a partial expression, however, baseline IFN-γ levels were very high and downstream STAT1 phosphorylation was found to be absent suggesting that although partial expression of IFN-γR1 was present, it was non-functional. All patients received ATT for mycobacterial infections. Two (25%) patients (P1 and P12) expired due to respiratory distress and hypersplenism with increased transfusion requirement. One patient (P54) with complete IFN-γR1 underwent haploidentical HSCT as the child had no matched family donor, nor any matched unrelated donor. He rejected his graft and had autologous reconstitution by day 28 post-HSCT. He continues to be on four ATT drugs. One patient (P35) is well on ATT and NTM prophylaxis. Four patients are (P21, P22, P31, and P48) are lost to follow up.

#### AD Partial IFN-γR1 Deficiency

**Figure 6:**
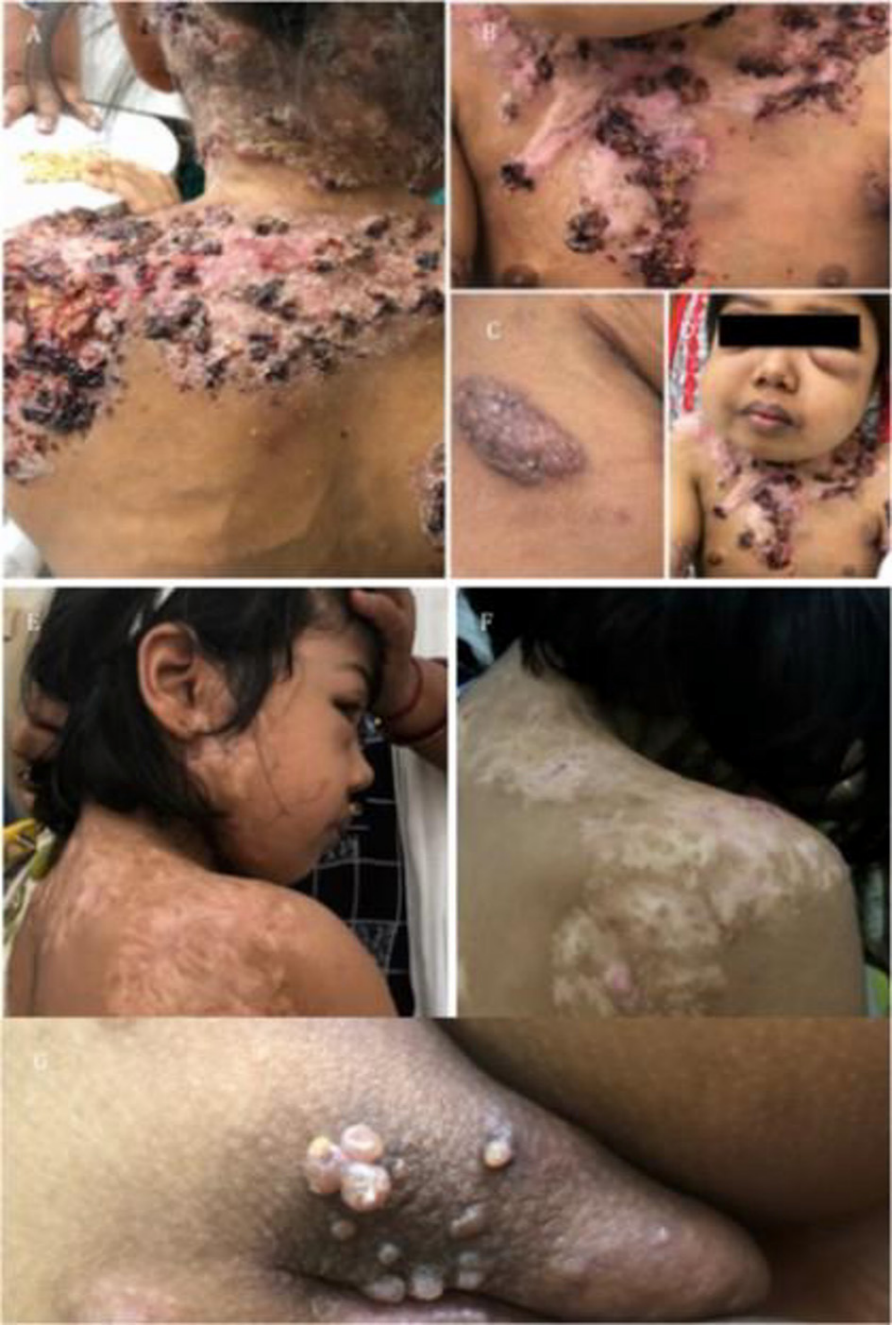
Clinical images of a case with partial dominant IFNγR1 deficiency. **(A–D)** Severe cutaneous and bony MAC infection. **(E,F)** Response to AKT. **(G)** Development of *Molluscum contagiosum*.

Seven patients were diagnosed with a partial dominant (PD) IFN-γR1 deficiency with a median age of presentation of 72 months (4–180 months). BCG-osis was the presenting manifestation in 6/7(86%) patients. Severe forms of the disease in the form of multifocal osteomyelitis were identified in (71%) five patients (P2, P11, P13, P44, and P47) with PD IFN-γR1 defect which was similar to world literature (21). The organisms isolated included tubercular and non-tubercular mycobacteria. One patient (P11) also identified with PD IFN-γR1defect had recurrent lymphadenopathy and joint involvement and extensive cutaneous infection with *Mycobacterium avium* intracellulare (Figure 6). She also developed molluscum contagiosum of the vulvar region. Other infections associated with partial dominant defect were *Pseudomonas aeruginosa* (P2), *Staphylococcus aureus* (P11), *Mycobacterium leprae* (P13), *Salmonella* (P44), *CMV* (P2) leading to secondary hemophagocytic lymphohistiocytosis (HLH), and *Varicella* (P27). Flow cytometry was performed in all seven patients, and showed increased IFN-γR1 expression, and abnormal STAT1 phosphorylation suggestive of the PD IFN-γR1 deficiency. All four patients in whom mutation was available had c.818_821delTTAA in exon 6 of *IFNGR1*. All patients received anti-tubercular treatment for mycobacterial infections. P27 needed three courses of ATT for recurrent lymphadenopathy. Two patients (P2 and P13) expired while two patients (P44 and P47) are lost to follow up. Rest three patients (P11, P23, and P27) are alive and doing well.

#### AR Complete IFN-γR2 Deficiency

IFN-γR2 deficiency was identified in eight patients (14%) which is higher than 3% reported in world literature (21). The median age of presentation is 6.5 months (3–14 months). All vaccinated patients presented with BCG-osis; two patients were not vaccinated due to previous affected sibling. One patient (P43) had a previously affected siblings, he had recurrent mycobacterial infection and needed three courses of ATT. Other infections identified included CMV infection in P51 and P52 and *Neisseria* in P52; both were siblings. Consanguinity was identified in 6/8 (75%) families. Three (37.5%) patients (P24, P29, and P38) expired before transplant. The siblings P51 and P52 underwent haplo-identical transplant; P51 is alive and well, while P52 expired 2 years later due to an unrelated cause.

#### STAT1 Deficiency

STAT1 deficiency was identified in four patients of whom two patients had an AR complete defect (P4 and P5) and two had a PD partial defect (P30 and P42). Both patients with a complete defect and one with a partial defect (P30) presented with BCG-osis and one patient with partial defect (P42) had history of recurrent tuberculosis. No other infections besides MTBC were identified in any patients. Both patients with complete defect were siblings and born of a consanguineous marriage. One patient with a partial defect (P30) had family history of father affected with cutaneous tuberculosis and paternal grandmother affected with Pott's spine, suggestive of autosomal dominant transmission. STAT1 phosphorylation by flow cytometry was found to be absent in both patients with complete STAT1 defect who were evaluated along with normal IFN-γ levels. Both patients with complete defect expired, one patient with PD defect (P42) is on antibiotic prophylaxis for recurrent respiratory tract infections and the other is on anti-mycobacterial treatment.

#### ISG15 Defect

One patient (P37) was identified with ISG15 defect, born of a third-degree consanguineous marriage had presented at 3 months of age with BCG-osis, he also had *Bocavirus* infection and recurrent Streptococcal pneumonia. A homozygous mutation was identified in exon 2 of the *ISG15* gene, c.454_455del, identified by NGS and confirmed by Sanger sequencing. The patient received ATT and antibiotics but succumbed to the disease.

#### RORγT Deficiency

One patient (P26) was identified with RORγT deficiency caused due to homozygous mutation in Exon 5 of the *RORC* gene, c.558T>G, identified by NGS and confirmed by Sanger sequencing by Dr. Anne Puel. The patient had presented with CNS tuberculomas, oral thrush, and onychomycosis. The patient received ATT and is currently well.

### Treatment and Outcome

All patients were started on ATT comprising of a combination of four drugs -isoniazid, rifampicin, ethambutol, and pyrazinamide, which was altered based on sensitivity pattern. BCG strain was identified based on its inherent pyrazinamide resistance [Danish 1331 is pyrazinamide resistant (11)] hence in BCG-osis, pyrazinamide was not part of treatment protocol. Duration of treatment was 6 months to 2 years depending on the site of involvement. Eighty-five percent received one course of ATT, 7% received two courses, 7% received three courses and 2% received five courses of ATT. Second-line drugs were given for a longer duration for MDR tuberculosis. Other antibiotics and antifungals based on drug sensitivity reports were added depending on concurrent infections. 6/51 (12%) cases of molecularly confirmed MSMD underwent allogenic HSCT. Three patients of complete IL-12Rβ1 (P32, P53, and P55) engrafted well and the patients are doing well. P32 and P53 had father as haploidentical donor while P55 had a fully matched sibling donor. One patient with complete IFN-γR1 (P54) underwent haploidentical HSCT as the child had no matched family donor, nor any matched unrelated donor. He rejected his graft and had autologous reconstitution by day 28 post-HSCT. He continues to be on four drug ATT. The siblings P51and P52 with complete IFN-γR2 deficiency underwent haplo-identical transplant from father; P51 is alive and well, while P52 expired 2 years later due to an unrelated cause. Three patients (P51, P52, and P53) were transplanted at Narayana Hrudayalaya, two (P54 and P55) at Apollo Chennai and One (P32) at CMC Vellore. Among 41 patients who could be followed 66% (27/41) are alive and well. Fourteen patients (34%) expired of which four had IL-12Rβ1 defect, two had AR complete IFN-γR1 deficiency, one had PD IFN-γR1 defect, four had AR complete IFN-γR2 defect, two AR complete STAT1 deficiency, and one AR ISG15 defect.

## Discussion

Although Inborn Errors of Immunity (IEI) as a group have been traditionally described to cause susceptibility to a wide range of micro-organisms, MSMD causes predisposition to infection by selective intracellular organisms. BCG related complications may be the presenting manifestation in MSMD in 4–80% of the patients (15). The practice of vaccinating all babies soon after birth might be the reason for an early presentation seen in our cohort; in addition to other well-known social factors like high population density, high incidence of *M. tb* in the country, poor nutrition, and unhygienic conditions. Although the majority of our patients presented at <3 years of age, some patients presented later in life and their diagnosis was confirmed after several years. Like other inherited genetic IEI, MSMD too can present at any age and should be kept in the differential diagnosis of any child with the right clinical infection to suspect MSMD. This was observed in particular in two patients where the diagnosis was delayed for years before the infection gave a clue to MSMD. One child (P8) was diagnosed after a lymph node biopsy grew non-typhi *Salmonella* which was the clue to suspect MSMD and IL-12Rβ1 defect was confirmed. Another child (P7) with BCG-osis after birth recovered and had two episodes of infection with *Histoplasma*; right shoulder osteomyelitis and later disseminated histoplasmosis. The clue to suspecting MSMD comes from knowing the organism. Local endemicity pattern for a particular infection (e.g., *Leishmania donovani, Histoplasma capsulatum*) may provide a difference in the pattern of infections in MSMD in different parts of the world. In India, *Leishmania donovani* is endemic in states of Bihar and Uttar Pradesh, however, we did not see a single case of MSMD with *Leishmania donovani* in our cohort while we did have cases of MSMD presenting with Histoplasmosis which is endemic in India.

Presence of mycobacterial infection in all patients suggests that the burden of exposure to *Mycobacterium* is very high in India. Isolation of NTM at a site other than the cervical node or wound site especially in the scenario of multifocal bone tuberculosis should initiate work up for underlying MSMD (23, 24). NTM was present in only 5% of our patients which is much less than what is reported in literature (25).

There is now a greater emphasis of making a definitive diagnosis of *M. tb* in India and due to the availability of culture, GeneXpert, line probe assay, and NGS for mycobacterial genome, it is possible to make a correct microbiological diagnosis. In addition to accurately identifying the organism, the drug sensitivity pattern can help us treat our patients more appropriately and recognize multi-drug resistant (MDR) and extensively drug-resistant (XDR) tuberculosis.

Micro-organisms isolated can provide very important clues to underlying molecular defects. For instance, an association of Mycobacterial infection with CMV strongly suggests AR complete IFNγR1/R2 or AR complete STAT1 defect (26); with *candidiasis* suggests possibility of IL-12Rβ1 defect or RORγT deficiency (27, 28); with *Salmonella* or non-typhi *Salmonella* strongly suggests the possibility of complete IL-12Rβ1 or IL-12p40 defect (29). Brain calcification along with mycobacterial infection suggests the possibility of ISG15 deficiency (30). Multifocal bone osteomyelitis by *M. tb* or NTM strongly correlates with an underlying molecular defect of PD-IFNγR1 defect or PD-STAT1 defect (31). One can predict an underlying molecular defect based on the clinical presentation.

Complete IL-12Rβ1 Deficiency caused by bi-allelic mutations in the *IL12RB1* gene was the commonest underlying molecular defect identified in our cohort. Mutations resulting in premature stop codons, such as nonsense, and essential splicing-site mutations, represented the majority of IL-12Rβ1 deficiency causing mutations (92%). This defect is usually associated with a late presentation, however, in our cohort, the median age of presentation was earlier at 9 months due to mandatory BCG vaccination at birth. The high incidence of nonsense and essential splice site mutation could also contribute to earlier presentation. Almost 64% of patients had a single infection with MTBC, this finding is consistent with the finding reported previously in a survey of 141 IL-12Rβ1 patients (32). It reflects the protective role of the primary infection against the reactivation of a latent organism or secondary infection. When compared to IFNγR1 defects and STAT1 deficiency, IL-12Rβ1 defect had a milder course of the disease which was observed by lower mortality (16% in IL-12Rβ1 compared to 20% in IFNγR1, and 50% in STAT1 defects altogether), fewer patients suffering from infections with multiple organisms, fewer courses of ATT required during the course of disease (32, 33). Although, the commonest mutation reported in literature is intron 15 c.1791+2T>G (34), we found exon 9 c.962C>A mutation to be the commonest in our cohort, which was present in almost half the patients. Multifocal bone tuberculosis may be seen in as much as half of the patients of PD IFNγR1 deficiency (17). This is consistent with the finding in our cohort (57%) with three patients having multifocal bone tuberculosis and one patient having joint involvement. STAT1 defects may also present with multifocal bone tuberculosis although this was not observed in our cohort.

Identification of a molecular defect can help us to take therapeutic management decisions. Patients with complete IFN-γR1, IFN-γR2, and STAT1 deficiencies can be cured with HSCT (35). Majority of our patients were treated with 4 drug ATT as is the usual practice in our country, the duration of treatment ranging from 6 months to 2 years depending on the site of involvement and the clinical response to treatment. However, since most of the patients had BCG (Danish 1,331) disease, which is resistant to pyrazinamide, standard ATT was modified to include drugs according to the sensitivity pattern and more aggressive treatment was given for a longer duration. A few patients of IL-12Rβ1 had a recurrence of infections and worsening of symptoms despite being on treatment. Such patients may benefit from treatment with IFN-γ (36) which is currently not available in India. HSCT was performed in three patients (P33, P54, and P56) with IL-12Rβ1 defect who are currently doing well.

This study describes the clinical and molecular spectrum of a large cohort of MSMD patients from India. It highlights the importance of having a high index of suspicion in patients presenting with adverse effects to BCG vaccination and investigating the IFN-γ mediated immunity in all patients with a clinical suspicion of MSMD (21) irrespective of age. Flow cytometric evaluation is helpful in rapid diagnosis and provides important clues to the underlying genetic defect. It is also helpful for functional validation of novel genetic defects (37). With the increasing burden of MDR and XDR TB in India, awareness about MSMD will help clinicians to evaluate more patients for underlying genetic susceptibility to mycobacteria. Under the national TB program (RNTCP) microbiological confirmation is mandatory before initiation of ATT. With increasing awareness among physicians isolation of NTM/ EM while pursuing the microbiological diagnosis would prompt work up for underlying genetic defects. Knowledge of the underlying molecular defect is important not only in planning definitive therapy in the form of HSCT for severe forms of the disease but also providing genetic counseling to the affected families. Once a family member has been diagnosed with MSMD, BCG vaccination should be avoided in the next child until a genetic defect has been ruled out.

Tuberculosis still remains a major public health problem in India. With BCG vaccine given at birth and tuberculosis being endemic in India, diagnosing MSMD is critical not only for the appropriate management of the patient but also for the optimum control of tuberculosis. As seen in our cohort BCG-osis is the commonest presentation. We had high index of suspicion for BCG-osis in patients presenting with BCG adenitis, persistent AFB positivity, three episodes of MTB, strong family history of Koch, infections with Intracellular organisms like Non-typhi salmonella, Kala Azar, Histoplasma, and NTM. This has helped us in picking up cases of MSMD and instituting appropriate treatment including HSCT. This study also highlights the wide spectrum of micro-organisms seen with MSMD which is unique to our cohort. We also report higher prevalence of IFNγR2 defect in India. Autoimmunity in MSMD has not been reported any literature, presence of multiple autoimmunity in our cohort of IL-12Rβ1 requires further investigation and may indicate need for screening MSMD patients for autoimmune diseases. Flow cytometric analysis of MSMD is available at only two centers in our country this has limited functional validation in our cohort.

## Data Availability Statement

The original contributions presented in the study are included in the article/supplementary material, further inquiries can be directed to the corresponding author/s.

## Ethics Statement

Ethical review and approval was not required for the study on human participants in accordance with the local legislation and institutional requirements. Written informed consent from the participants' legal guardian/next of kin was not required to participate in this study in accordance with the national legislation and the institutional requirements. Written informed consent was obtained from the minor(s)' legal guardian/next of kin for the publication of any potentially identifiable images or data included in this article.

## Author Contributions

PT, MD, and RY compiled the data, wrote, and edited the manuscript. VG, AAP, VVI, AC, ZG, SC, RA, PK, ND, BG, NF, EE, AA, AR, JD, VJ, RP, AJ, SuB, SaB, JU, NR, RR, RU, SP, HL, AA, MK, ZU, VB, and TK provided patient data and conducted clinical exploration and treatment of subjects. AD, UB, and PMK did the flow work up of MSMD cases. JB, JC, and AP provided the molecular analysis. MD, MM, SSP, and MB supervised the study, reviewed, and approved the final version of the manuscript. All authors contributed to the article and approved the submitted version.

## Conflict of Interest

The authors declare that the research was conducted in the absence of any commercial or financial relationships that could be construed as a potential conflict of interest.

## References

[1] Rosain J Kong X Martinez-Barricarte R Oleaga-Quintas C Ramirez-Alejo N Markle J. Mendelian susceptibility to mycobacterial disease: 2014–2018 update. Immunol Cell Biol. (2019) 97:360–7. 10.1111/imcb.12210 30264912 PMC6438774

[2] Casanova J-L Abel L. Genetic dissection of immunity to mycobacteria: the human model. Annu Rev Immunol. (2002) 20:581–620. 10.1146/annurev.immunol.20.081501.125851 11861613

[3] Tangye SG Al-Herz W Bousfiha A Chatila T Cunningham-Rundles C Etzioni A. Human inborn errors of immunity: 2019 update on the classification from the International Union of Immunological Societies Expert Committee. J Clin Immunol. (2020) 40:24–64. 10.1007/s10875-019-00737-x 31953710 PMC7082301

[4] MacLennan C Fieschi C Lammas DA Picard C Dorman SE Sanal O. Interleukin (IL)-12 and IL-23 are key cytokines for immunity against Salmonella in humans. J Infect Dis. (2004) 190:1755–7. 10.1086/425021 15499529

[5] Wu UI Holland SM. A genetic perspective on granulomatous diseases with an emphasis on mycobacterial infections. Semin Immunopathol. (2016) 38:199–212. 10.1007/s00281-015-0552-y 26733044 PMC4779418

[6] Ouederni M Sanal O Ikinciogullari A Tezcan I Dogu F Sologuren I. Clinical features of candidiasis in patients with inherited interleukin 12 receptor β1 deficiency. Clin Infect Dis. (2014) 58:204–13. 10.1093/cid/cit722 24186907 PMC3871796

[7] León-Lara X Hernández-Nieto L Zamora CV Rodríguez-D'Cid R Gutiérrez MEC Espinosa-Padilla S. Disseminated infectious disease caused by histoplasma capsulatum in an adult patient as first manifestation of inherited IL-12Rβ1 deficiency. J Clin Immunol. (2020) 40:1051–4. 10.1007/s10875-020-00828-0 32710397

[8] Parvaneh N Barlogis V Alborzi A Deswarte C Boisson-Dupuis S Migaud M Farnaria C. Visceral leishmaniasis in two patients with IL-12p40 and IL-12Rβ1 deficiencies. Pediatr Blood Cancer. (2017) 64:e26362. 10.1002/pbc.26362 27873456

[9] Dorman SE Uzel G Roesler J Bradley JS Bastian J Billman G. Viral infections in interferon-γ receptor deficiency. J Pediatr. (1999) 135:640–3. 10.1016/S0022-3476(99)70064-8 10547254 PMC7095028

[10] Ministry of Health & family Welfare, Central Tuberculosis Division Government of India. National Tuberculosis elimination Programme Annual Report [Internet]. New Delhi: Government of India (2020). Available online at: https://tbcindia.gov.in/showfile.php?lid=3538

[11] World Health Organization. BCG vaccine: WHO position paper, February 2018 – recommendations. Vaccine. (2018) 36, 3408–10. 10.1016/j.vaccine.2018.03.009 29609965

[12] Bandari AK Muthusamy B Bhat S Govindaraj P Rajagopalan P Dalvi A. A novel splice site mutation in IFNGR2 in patients with primary immunodeficiency exhibiting susceptibility to mycobacterial diseases. Front Immunol. (2019) 10:1964. 10.3389/fimmu.2019.01964 31497017 PMC6712061

[13] Merchant RH Ahmed J Ahmad N. XDR TB in a case of IL12Rβ1 deficiency: a case report of mendelian susceptibility to mycobacterial disease from India. Indian J Pediatr. (2013) 80:781–2. 10.1007/s12098-012-0806-9 22696093

[14] Sharma VK Pai G Deswarte C Lodha R Singh S Kang LW. Disseminated *Mycobacterium avium* complex infection in a child with partial dominant interferon gamma receptor 1 deficiency in India. J Clin Immunol. (2015) 35:459–62. 10.1007/s10875-015-0173-1 26054576

[15] Indumathi CK Kowtal PM Poornima RN Lewin S. Clinical profile and outcome of clinical BCG disease in infants. Indian Pediatr. (2014) 51:730–2. 10.1007/s13312-014-0491-z 25228607

[16] Bernatowska EA Wolska-Kusnierz B Pac M Kurenko-Deptuch M Zwolska Z Casanova J-L. Disseminated bacillus Calmette-Guérin infection and immunodeficiency. Emerg Infect Dis. (2007) 13:799. 10.3201/eid1305.060865 18044052 PMC2738440

[17] Nekooie-Marnany N Deswarte C Ostadi V Bagherpour B Taleby E Ganjalikhani-Hakemi M. Impaired IL-12- and IL-23-mediated immunity due to IL-12Rβ1 deficiency in Iranian patients with mendelian susceptibility to mycobacterial disease. J Clin Immunol. (2018) 38:787–93. 10.1007/s10875-018-0548-1 30255293 PMC6469360

[18] Lim AI Menegatti S Bustamante J Le Bourhis L Allez M Rogge L. IL-12 drives functional plasticity of human group 2 innate lymphoid cells. J Exp Med. (2016) 213:569–83. 10.1084/jem.20151750 26976630 PMC4821648

[19] van de Vosse E van Dissel JT. IFN-γR1 defects: mutation update and description of the IFNGR1 variation database. Hum Mutat. (2017) 38:1286–96. 10.1002/humu.23302 28744922

[20] Jouanguy E Dupuis S Pallier A Döffinger R Fondanèche MC Fieschi C. In a novel form of IFN-γ receptor 1 deficiency, cell surface receptors fail to bind IFN-γ. J Clin Invest. (2000) 105:1429–36. 10.1172/JCI9166 10811850 PMC315467

[21] Bustamante J Boisson-Dupuis S Abel L Casanova J-L. Mendelian susceptibility to mycobacterial disease: Genetic, immunological, and clinical features of inborn errors of IFN-γ immunity. Semin Immunol. (2014) 26, 454–70. 10.1016/j.smim.2014.09.008 25453225 PMC4357480

[22] BustamanteJ. Mendelian susceptibility to mycobacterial disease: recent discoveries. Hum Genet. (2020) 139:993–1000. 10.1007/s00439-020-02120-y 32025907 PMC7275902

[23] Mandell DL Wald ER Michaels MG Dohar JE. Management of nontuberculous mycobacterial cervical lymphadenitis. Arch Otolaryngol Neck Surg. (2003) 129:341–4. 10.1001/archotol.129.3.341 12622546

[24] Lake MA Ambrose LR Lipman MCI Lowe DM. “Why me, why now?” Using clinical immunology and epidemiology to explain who gets nontuberculous mycobacterial infection. BMC Med. (2016) 14:1–13. 10.1186/s12916-016-0606-6 27007918 PMC4806462

[25] Ratnatunga CN Lutzky VP Kupz A Doolan DL Reid DW Field M. The rise of non-tuberculosis mycobacterial lung disease. Front Immunol. (2020) 11:303. 10.3389/fimmu.2020.00303 32194556 PMC7062685

[26] Casanova JL Ochs HD. Interferon-γ receptor deficiency: an expanding clinical phenotype? J Pediatr. (1999) 135:543–5. 10.1016/S0022-3476(99)70050-8 10547240

[27] Okada S Markle JG Deenick EK Mele F Averbuch D Lagos M. Impairment of immunity to Candida and *Mycobacterium* in humans with bi-allelic RORC mutations. Science. (2015) 349:606–13. 10.1126/science.aaa4282 26160376 PMC4668938

[28] De Beaucoudtey L Puel A Filipe-Santos O Cobat A Ghandil P Chrabieh M. Mutations in STAT3 and IL12RB1 impair the development of human IL-17-producing T cells. J Exp Med. (2008) 205:1543–50. 10.1084/jem.20080321 18591412 PMC2442631

[29] Tan Ç Çagdaş-Ayvaz D Metin A Keskin Ö Tezcan I Sanal Ö. Clinical and genetic features of IL12Rβ1 deficiency: single center experience of 18 patients. Turk J Pediatr. (2016) 58:356–61. 10.24953/turkjped.2016.04.002 28276206

[30] Zhang X Bogunovic D Payelle-Brogard B Francois-Newton V Speer SD Yuan C. Human intracellular ISG15 prevents interferon-α/β over-amplification and auto-inflammation. Nature. (2015) 517:89–93. 10.1038/nature13801 25307056 PMC4303590

[31] Nishimura S Tsumura M Hirata O Kagawa R Mizoguchi Y Okada S. MSMD patients with IFN-g-STAT1 signaling defect present enhanced osteoclastogenesis and bone resorption. Blood. (2015) 126:3591. 10.1182/blood.V126.23.3591.3591

[32] De Beaucoudrey L Samarina A Bustamante J Cobat A Boisson-Dupuis S Feinberg J. Revisiting human IL-12Rβ1 deficiency: a survey of 141 patients from 30 countries. Medicine. (2010) 89:381. 10.1097/MD.0b013e3181fdd832 21057261 PMC3129625

[33] Fieschi C Dupuis S Catherinot E Feinberg J Bustamante J Breiman A. Low penetrance, broad resistance, and favorable outcome of interleukin 12 receptor β1 deficiency: Medical and immunological implications. J Exp Med. (2003) 197:527–35. 10.1084/jem.20021769 12591909 PMC2193866

[34] van de Vosse E Haverkamp MH Ramirez-Alejo N Martinez-Gallo M Blancas-Galicia L Metin A. IL-12Rβ1 deficiency: mutation update and description of the IL12RB1 variation database. Hum Mutat. (2013) 34:1329–39. 10.1002/humu.22380 23864330 PMC4104692

[35] Tovo P-A Garazzino S Saglio F Scolfaro C Bustamante J Badolato R. Successful hematopoietic stem cell transplantation in a patient with complete IFN-γ receptor 2 deficiency: a case report and literature review. J Clin Immunol. (2020) 40:1191–5. 10.1007/s10875-020-00855-x 32909233 PMC7567729

[36] Bax HI Freeman AF Ding L Hsu AP Marciano B Kristosturyan E. Interferon alpha treatment of patients with impaired interferon gamma signaling. J Clin Immunol. (2013) 33:991–1001. 10.1007/s10875-013-9882-5 23512243 PMC4136390

[37] Esteve-Solé A Sologuren I Martínez-Saavedra MT Deyà-Martínez À Oleaga-Quintas C Martinez-Barricarte R. Laboratory evaluation of the IFN-γ circuit for the molecular diagnosis of Mendelian susceptibility to mycobacterial disease. Crit Rev Clin Lab Sci. (2018) 55:184–204. 10.1080/10408363.2018.1444580 29502462 PMC5880527

